# Purified Vitexin Compound 1 Inhibits UVA-Induced Cellular Senescence in Human Dermal Fibroblasts by Binding Mitogen-Activated Protein Kinase 1

**DOI:** 10.3389/fcell.2020.00691

**Published:** 2020-07-31

**Authors:** Ben Wang, Sha Yan, Yuxin Yi, Yingxue Huang, Zhili Deng, Yiya Zhang, Qingchuan Zheng, Hongfu Xie, Ji Li

**Affiliations:** ^1^Department of Dermatology, Xiangya Hospital, Central South University, Changsha, China; ^2^National Clinical Research Center for Geriatric Disorders, Xiangya Hospital, Central South University, Changsha, China; ^3^Laboratory of Theoretical and Computational Chemistry, Institute of Theoretical Chemistry, Jilin University, Changchun, China; ^4^Key Laboratory of Organ Injury, Aging and Regenerative Medicine of Hunan Province, Changsha, China; ^5^Center for Molecular Medicine, Xiangya Hospital, Central South University, Changsha, China

**Keywords:** purified vitexin compound 1, VB1, senescence, skin photoaging, MAPK1

## Abstract

Purified vitexin compound 1 (VB1), a novel lignanoid isolated from the seeds of the Chinese herb Vitex negundo, has strong antioxidant abilities and broad antitumor activities. However, little is known about its anti-photoaging effect on the skin and the underlying mechanism. Here, we demonstrated that VB1 significantly attenuates ultraviolet A (UVA)-induced senescence in human dermal fibroblasts (HDFs), as evidenced by senescence-associated β-gal staining, MTT assays, and western blot analysis of the expression of p16 and matrix metalloproteinase-1 (MMP-1). Furthermore, mass spectrometry revealed that VB1 could directly bind to Mitogen-Activated Protein Kinase 1 (MAPK1). Molecular docking and molecular dynamics simulation methods confirmed the mass spectroscopy results and predicted six possible binding amino acids of MAPK1 that most likely interacted with VB1. Subsequent immunoprecipitation analysis, including different MAPK1 mutants, revealed that VB1 directly interacted with the residues, glutamic acid 58 (E58) and arginine 65 (R65) of MAPK1, leading to the partial reversal of UVA-induced senescence in HEK293T cells. Finally, we demonstrated that the topical application of VB1 to the skin of mice significantly reduced photoaging phenotypes *in vivo*. Collectively, these data demonstrated that VB1 reduces UVA-induced senescence by targeting MAPK1 and alleviates skin photoaging in mice, suggesting that VB1 may be applicable for the prevention and treatment of skin photoaging.

## Introduction

Chronic exposure to ultraviolet (UV) irradiation is the major cause of skin damage leading to premature aging of the skin, a condition called photoaging (Kruglikov and Scherer, 2016). Clinical changes in the course of skin photoaging include the formation of fine and coarse wrinkles, increased skin thickness, dryness, laxity, and pigmentation (Lei et al., 2018). Solar UV radiation is divided into three categories according to their wavelength. UV radiation can penetrate the skin to different extents and interact with skin cells (Bravo et al., 2017). UVA (320–400 nm) is more abundant in sunlight and penetrates the skin deeper than UVB (280–315 nm). Previous studies have revealed that UVA plays an important role in skin photoaging (Kammeyer and Luiten, 2015; Lei et al., 2018).

To date, the mechanisms of skin photoaging is still unknown, however, it is mainly associated with oxidative stress, inflammatory responses, and DNA damage (Shin et al., 2019). Oxidative stress can increase the secretion of proteases and produce a large number of oxidative intermediates due to an imbalance between the production of oxidants and antioxidants. Accumulation of reactive oxygen species (ROS) induced by oxidative stress can affect skin cells in both epidermis and dermis, promoting cellular senescence (Stellavato et al., 2018). Oxidative stress, one of the most important mechanisms underlying skin photoaging, activates the Mitogen Activated Protein Kinase (MAPK) family, including extracellular signal-regulated protein kinases1/2 (ERK 1/2), c-Jun NH2-terminal kinase (JNK or SAPK), and p38 MAPK, and their downstream pathways to promote cellular senescence (Sun et al., 2015). MAPK1, also called ERK2, is one of the key molecules in signal transduction pathways associated with cellular senescence and only functional when phosphorylated. Recent studies revealed that MAPK1 plays a major role in the unbalanced growth of human cells (Kobayashi et al., 2012). Vitamin D protects endothelial cells from irradiation-induced senescence and apoptosis by modulating the MAPK/Sirtuin 1 (SirT1) axis (Marampon et al., 2016). Naringenin exerts potent anti-photoaging effects by suppressing UVB-induced phosphorylated MAPK1 activity in JB6 P + cells, indicating that MAPK1 plays an important role in the cellular senescence (Jung et al., 2016). In addition, some other well-studied genes act as aging markers that are often studied: Meis1 is a putative regulators of neurotransmission and neurogenesis during aging (Chang-Panesso et al., 2018). Rb1, another aging marker, induces senescence in human skin fibroblasts by regulating by DNA methyltransferase 1 (DNMT1) (Wang et al., 2017).

Purified vitexin compound 1 (VB1), a novel lignanoid isolated from the seeds of the Chinese herb, *Vitex negundo*, has strong antioxidant abilities and broad antitumor activities in many cancer cell lines and xenograft models (Liu et al., 2018). VB1 suppresses the growth of melanoma cells and induces apoptosis in breast cancer cells by increasing the ROS level (Liu et al., 2014). However, VB1 failed to induce ROS generation in the immortalized non-cancerous breast cell line, indicating that it has different effects on oxidative stress processes depending on cellular conditions. In addition, VB-1 can exert hair growth-promoting effects by augmenting Wnt/β-catenin signaling in human dermal papilla cells and protect PC12 cells from hypoxia/reoxygenation-induced injury via NADPH oxidase inhibition (Yang et al., 2014; Luo et al., 2018). However, little is known about the role of VB1 in skin photoaging. Considering that oxidative stress is an important mechanism of skin photoaging, and VB1 an antioxidative agent, we speculated that VB1 may play an important role in skin photoaging.

In this study, we found that VB1 significantly inhibited UVA-induced senescence in human dermal fibroblasts (HDFs). Using mass spectrometry, we also revealed that VB1 could directly bind to MAPK1. Computer-aided methods and immunoprecipitation demonstrated that VB1 binds to MAPK1 in 293T cells by interacting with the residues E58 and R65. We further verified that VB1 partially reverses UVA-induced senescence by the above-mentioned binding to MAPK1. Finally, topical VB1 gel remarkably reduced the phenotype of skin photoaging in mice. For the first time, we demonstrate that VB1 reduces UVA-induced senescence in HDFs by targeting the residues E58 and R65 in MAPK1 and alleviates skin photoaging in mice. Our results indicate that VB1 is a potential new drug for the prevention and treatment of skin photoaging in the future.

## Materials and Methods

### Reagents

VB1 [vitexin compound-1, 6-hydroxy-4-(4-hydroxy-3-methoxyphenyl)-3-hydroxymethyl-7-methoxy-3,4-dihydro-2-na phthaldehyde] was a kind gift from the School of Pharmacy at Central South University (Changsha, Hunan, China). The gel containing 2% VB1 or the vehicle gel lacking VB1 were also given from the School of Pharmacy at Central South University. Primary antibodies specific for human against MMP-1, p16, MAPK1, and p-MAPK1 were purchased from Cell Signaling Technology (Boston, MA, United States).

### Cell Culture

Primary HDFs were isolated from circumcised foreskins of healthy human donors aged from 5 to 12 years. Primary HDFs were cultured at 37°C and 5% CO_2_ in a humidified incubator in Dulbecco’s modified Eagle media (DMEM; Gibco, Grand Island, NY, United States), supplemented with penicillin (100 U/mL), streptomycin (100 ng/mL), and 10% fetal bovine serum (FBS; Gibco). Primary HDFs were obtained with written consent from voluntary, informed donors, following a protocol approved by the Clinical Research Ethics Committee at the Xiangya Hospital of Central South University in Changsha, China.

### UVA Irradiation

Before UVA irradiation, HDFs cells were rinsed and submerged under a thin layer of PBS to prevent UVA absorption by components of the medium, such as VB1. Cells were then irradiated using a Philips UVA lamp with an emission spectrum between 320 and 400 nm. Mock-irradiated cells were manipulated identically, except that they were not exposed to UVA. The dose of UVA irradiation was 10 J/cm^2^ per day, as verified with a UV light meter (Sigma, Shanghai, China) for 3 days. Following each UVA irradiation, cells were incubated in complete medium, supplemented with indicated compounds.

### Western Blotting

Thirty micrograms of protein from each cell lysate was resolved by 10% SDS-PAGE, followed by electrotransfer to PVDF membranes (Millipore, MA, United States). Blots were probed with primary antibodies at 4°C overnight, followed by incubation with an HRP-conjugated secondary antibody for 1 h at room temperature. Bands of interest in western blots were visualized with a western blot HRP substrate (Millipore, Billerica, MA, United States).

### SA β-Gal Staining

Senescence-associated β-galactosidase (SA-β-gal) activity was measured with a β-galactosidase staining kit (Cell Signaling Technology Boston, MA, United States) according to the manufacturer’s instructions. Briefly, cells were washed in PBS, fixed at room temperature for 15 min in fixing solution, and incubated overnight at 37°C in staining solution. Relative SA-β-gal activities under each studied condition were determined by calculating the percentages of cells with SA-β-gal activity out of all cells counted in four continuous visual fields under a microscope (200x).

### MTT Assays

Cell viabilities were determined by performing 3-(4,5-dimethylthiazol-2-yl)-2,5 -diphenyltetrazoliumbromide (MTT) assays. Briefly, cells were seeded into 96-well plates at a density of 2,000 cells/well. After adhesion, cells were exposed to UVA irradiation and grown in complete medium containing VB1 (0.6 μM). At 1, 3, or 5 days post-irradiation, the medium was aspirated, and cells were incubated for 4 h in fresh medium containing 0.5 mg/mL of MTT (Sigma, St. Louis, MO, United States). Subsequently, the medium was removed and purple formazan crystals were dissolved in DMSO (150 μl/well) with a brief vortexing step. Absorbance at 570 nm was measured using a Synergy 2 Multi-Mode Microplate Reader (BioTek, Seattle, United States). All experiments were performed in triplicate, and the data presented represent the means of 3 independent experiments ± SD.

### Molecular Docking and Molecular Dynamics Simulation

The crystal structure of the wild type MAPK1 protein was obtained from Protein Data Bank (PDB code: 5BVF) (Bagdanoff et al., 2015). The missing residues and atoms were repaired by software Discovery Studio 2.5 (BIOVIA, CA, United States). The MAPK1-VB1 binding site was predicted by Discovery Studio 2.5 and Autodock Vina (Trott and Olson, 2010). The molecular docking study was performed employing the program Autodock Vina. And the Molecular dynamics (MD) simulations were performed to explore the binding details base the docking results. The partial atomic charge of VB1 was assigned by AM1-BCC method (Wang et al., 2006) and the topology files of VB1 were generated by AMBER force field (GAFF) (Wang et al., 2004). The protonation states of ionizable residues were determined at pH = 7.0 using H++ server (Gordon et al., 2005). The MD simulations were carried out using the AMBER 16 software package (Alma Rosa Agorilla, University of California, San Francisco, United States). First, 10,000 steps minimization (4,000 steps of steepest decent followed by 6,000 steps of conjugate gradient) was carried out with protein and inhibitor constrained (100 kcal mol-1 Å-2). Subsequently, the minimization was repeated with no constrain. Then, the system was gradually heated from 0 to 310 K over a period of 300 ps with 5.0 kcal mol-1 Å-2 restrain on the solute. Thereafter, another 1 ns equilibrium simulation was followed at 310 K with 2.0 kcal mol-1 Å-2 restrain on the solute. Finally, 100 ns MD simulation was performed for each system under NPT condition to produce trajectory. The time step was set to 2 fs.

### Synthesis and Modification of Gold Nanoparticles

Gold nanoparticles were synthesized according to following procedures. Briefly, 3 mL sodium citrate (w/v, 2%) was added to 100 mL boiling HAuCl4 (0.01%) solution and kept heated for 10 min. With continuous stirring until cooled to room temperature, the gold nanoparticles were synthesized. To conjugate lignin onto gold nanoparticles, cysteamine was first linked onto gold nanoparticles through the thiol group on the cysteamine to introduce amino group onto gold nanoparticles. Cysteamine was added into gold nanoparticles (final concentration of cysteamine is 10 M) and reacted for 2 h. After centrifuge, the gold nanoparticles were reacted with 1-(3-Dimethylaminopropyl)-3-ethylcarbodiimide hydrochloride (EDC, 100 mM) and N-hydroxysuccinimide (NHS, 100 mM) for 30 min. Then, lignin was added into the solution to reach a concentration of 10 g/mL. After another 2 h of reaction, the gold nanoparticles were centrifuged and re-dispersed into water for further use.

### HPLC – Mass Spectrometry Analysis

Each sample of enriched nanogold-VB1 compound was reconstituted in 7 μl of HPLC buffer A (0.1%(v/v) formic acid in water), and 5 μl was injected into a Nano-LC system (EASY-nLC 1000, Thermo Fisher Scientific, Waltham, MA, United States). Each sample was separated by a C18 column (50 μm inner-diameter × 15 cm, 2 μm C18) with a 125 min HPLC-gradient. The mass spectrometric analysis was carried out in a data-dependent mode with an automatic switch between a full MS scan and an MS/MS scan in the orbitrap. The resulting MS/MS data were searched against UniProt P. mirabilis ATCC 29906 database using MaxQuant software (v1.5.2.8).

### Generation of MAPK1 Mutants

The plasmid MAPK1-pENTER was purchased from vigenebio (Shandong, China). Mutants of MAPK1 were generated with a QuickChange II XL Site-Directed Mutagenesis Kit (Agilent Technologies, Palo Alto, CA, United States) according to the manufacturer’s instructions. The primers used for the Mutants were used were shown in Supplementary Table 1.

### Immunoprecipitation

HEK293 cells were transfected with MAPK1 or mut-MAPK1. The cells were collected and washed with ice-cold PBS and lysed in buffer, and the VB1 modified by gold nanoparticles was added and incubation continued overnight at 4°C. Precipitates were washed three times with ice-cold lysis buffer at 400 g for 10 min. Bound proteins were separated on an SDS – polyacrylamide gel and analyzed by western blotting using the anti-MAPK1 antibodies.

### Animals and UVA Radiation

Eight-week-old female FVB mice were obtained from the National Key Laboratory of Genetics (Changsha, Hunan, China). Animals were housed at 23 ± 1°C and 50 ± 10% relative humidity in a specific pathogen-free environment. Animal experiments were approved by the Animal Research Committee at the Xiang Ya Hospital of Central South University. The dorsal skin area of mice was shaved before and during experiments. Mice were divided into control, UVA,vehicle gel and VB1 groups, with 10 mice in each group. All Mice except control group were irradiated 3 times/week for 12 weeks with 20 J/cm^2^ doses under a Philips UVA lamp placed 20 cm away (emission spectrum: 320–400 nm). The dorsal skin of mice was washed with 75% ethanol before each irradiation exposure to avoid blocking or absorption of UVA rays by previous applications of the VB1 gel. UVA doses were verified with a UV light meter. A Carbomer substrate gel containing 2% VB1 or vehicle gel lacking VB1 was applied dorsally to the mice accordingly every day. No topical application or irradiation was performed in the control group.

### Histological Analysis

Mice were sacrificed by cervical dislocation under chloral hydrate anesthesia at the end of experiments. For histological analyses, central dorsum skin specimens were fixed in 4% paraformaldehyde and sectioned after paraffin embedding. Hematoxylin-eosin staining and Masson-trichrome staining was then performed. Photographs of 5 randomly-chosen fields in each section were taken under a microscope (200x). Epidermal thicknesses were measured as the distance from the basement membrane to the bottom of the stratum corneum.

### Statistical Analysis

All data presented are representative of at least 3 independent experiments and are expressed as means ± SD. Statistical significances were determined by a one-way analysis of variance, followed by further analysis by the LSD (least significant difference) test. *P* < 0.05 was considered statistically significant.

## Results

### VB1 Protects HDFs From UVA-Induced Senescence

**FIGURE 1:**
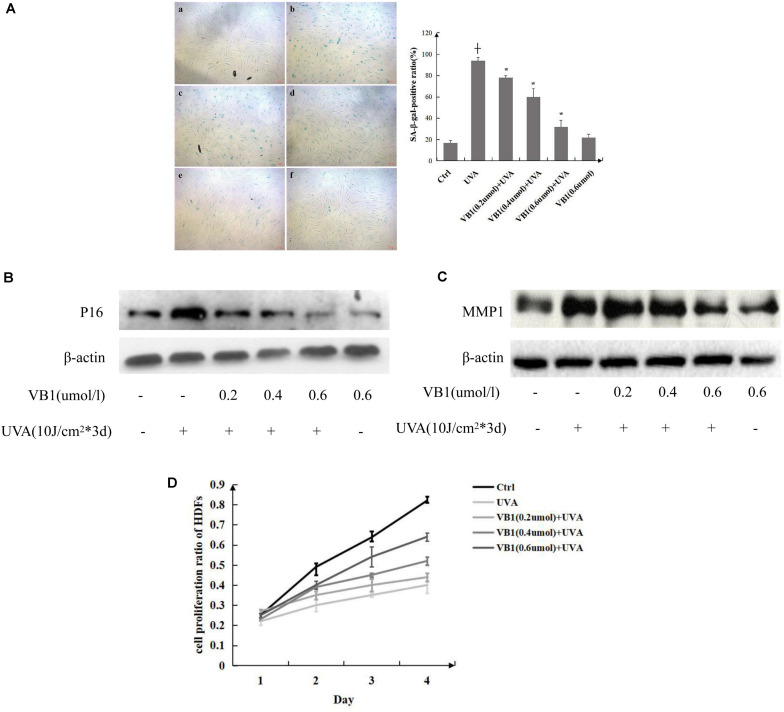
VB1 protects HDFs from UVA-induced senescence. **(A)** HDFs senescence was evaluated by measuring the number of SA-β-gal-positive cells (left panel) [a, Ctrl; b, UVA; c, VB1 (0.2 μmol/l) + UVA; d, VB1 (0.4 μmol/l) + UVA; e, VB1 (0.6 μmol/l) + UVA; f, VB1 (0.6 μmol/l)]. Percentages of SA-β-gal-positive cells were determined by counting stained cells and total cells in four continuous visual fields under a microscope (200x). The SA-β-gal-positive rate was obviously enhanced in UVA-induced HDFs, while VB1 inhibited UVA-induced SA-β-gal activity in a dose-dependent manner. The analysis data was shown in right panel. Data are presented as mean HDFs ± SD (*n* = 3; + vs. ctrl, *p* < 0.05, * vs UVA, *p* < 0.05). **(B)** p16 levels were detected by western blot analysis. Cells were irradiated as described and harvested 24 h after the final UVA exposure. Blots were probed to detect p16, stripped, and then reprobed for β-actin. VB1 inhibited UVA-induced p16 expression in a dose-dependent manner. Images are representative of 3 independent experiments. **(C)** MMP1 levels were detected by western blot analysis. VB1 inhibited UVA-induced MMP1 expression in a dose-dependent manner. Images are representative of 3 independent experiments. **(D)** The HDFs growth rate was determined by performing MTT assays. The growth rate of UVA-exposed HDFs was significantly decreased compared with control HSFs, while VB1 could reverse the decrease (*n* = 3 for each time point).

SA-β-gal activity was measured in HDFs to investigate the effects of VB1 on cellular senescence induced by UVA. Our results showed that the percentage of senescent cells (SA-β-gal-positive cells) was significantly increased in UVA-irradiated HDFs compared with that in non-irradiated control cells. VB1 inhibited UVA-induced SA-β-gal activity in a dose-dependent manner (Figure 1A). Additionally, we found that the expression of p16, a hallmark of cellular senescence, was significantly increased after UVA irradiation and that VB1 showed dose-dependent inhibition of p16 expression (Figure 1B). Previous studies have shown that UVA irradiation causes photoaging through MMP-1 induction, and MMP1 was also considered as an indicator of senescence-Associated Secretory Phenotype (Gorgoulis et al., 2019). Thus, MMP-1 expression was analyzed by western blotting to examine whether VB1 regulates its expression following UVA exposure. The results confirmed that UVA irradiation increased MMP-1 protein expression in HDFs, however, the UVA-induced induction of MMP-1 expression was inhibited by VB1 in a dose-dependent manner (Figure 1C). By MTT assays, we showed that UVA irradiation decreased the cell proliferation of HDFs and that VB1 treatment partially reversed this UVA-induced effect (Figure 1D). These data indicated that VB1 partially protected HDFs from UVA-induced senescence.

### Potential Target Proteins and Target Sites of VB1 for MAPK1

**FIGURE 2:**
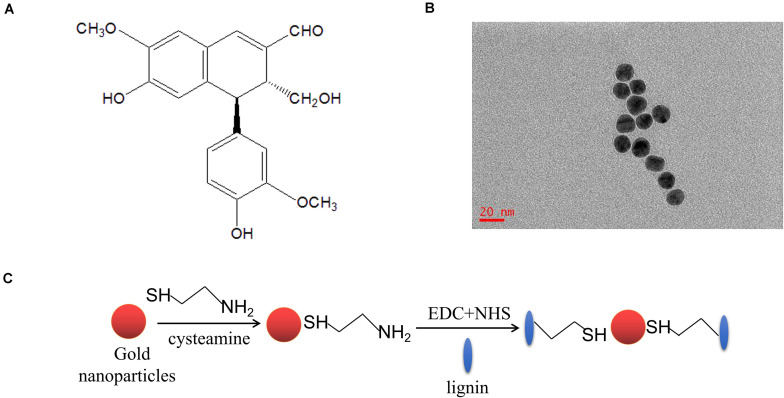
VB1 is linked with modified gold nanoparticles for further HPLC and immunoprecipitation. **(A)** The chemical structure diagram of VB1. **(B)** The gold nanoparticles were synthesized and shown in the transmission electron microscope (TEM) image. **(C)** Gold nanoparticles was modified by lignin via reacting with 1-(3-Dimethylaminopropyl)-3-ethylcarbodiimide hydrochloride (EDC, 100 mM) and N-hydroxysuccinimide (NHS, 100 mM).

**FIGURE 3:**
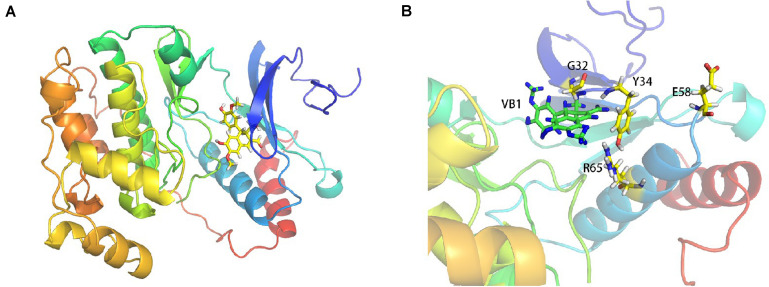
The possible target sites of MAPK1 binding VB1 are predicted by computer-aided methods including molecular docking and molecular dynamics simulation. The MAPK1-VB1 binding site was predicted by Discovery Studio 2.5 and Autodock Vina. The molecular docking study and the Molecular dynamics (MD) simulations were performed to explore the binding details base the docking results. **(A)** The molecular docking and molecular dynamics simulation results showed VB1 could directly bind MAPK1 protein. **(B)** The five amino acid residues (G32, Y34, K46, E58, and R65) of MAPK1 were predicted and K338 residue was another possible key residue through computer-aid calculation (data not shown).

To screen the potential binding target proteins of VB1, we used mass spectrometry. For this purpose, VB1 was linked to modified gold nanoparticles (Figure 2). Table 1 shows the potential target proteins of VB1 identified by mass spectrometry. Based on previous research on senescence, we chose MAPK1 as the target protein of VB1 for further experiments. To accurately predict the possible target sites of VB1 for MAPK1, we employed computer-aided methods, including molecular docking and molecular dynamics simulation were employed. The *in silico* results showed that VB1 could directly bind MAPK1 (Figure 3A), and we finally predicted that five likely interacting amino acid residues (G32, Y34, K46, E58, and R65) of MAPK1 (Figure 3B), and K338 was another possible key residue.

**TABLE 1:** The 26 possible target proteins of VB1.

The abbreviation of proteins	Proteins full names
MPRIP	Myosin phosphatase Rho-interacting protein
FLII	Flightless-1 homolog
ANPEP	Alanyl aminopeptidase
FLOT2	Flotillin 2
SERPINH1	Serpin family H member 1
ARHGAP23	Rho GTPase-activating protein 23
EIF2S1	Eukaryotic translation initiation factor 2 subunit 1
TWF1	Twinfilin actin binding protein 1
NPM1	Nucleophosmin 1
EEF1A	Elongation factor 1-alpha
ATP6V0D1	ATPase H^+^ transporting V0 subunit d1
HNRNPA3	Heterogeneous nuclear ribonucleoprotein A3
TPM1	Tropomyosin 1
NEFH	Neurofilament heavy polypeptide
MAPK1	Mitogen-activated protein kinase 1
CAPZB	Capping actin protein of muscle Z-line subunit beta
YWHAQ	Tyrosine 3-monooxygenase/tryptophan 5-monooxygenase activation protein theta
YWHAZ	Tyrosine 3-monooxygenase/tryptophan 5-monooxygenase activation protein zeta
ZNF366	Zinc finger protein 366
SLC25A1	Solute carrier family 25 member 1
DPM1	Dolichol-phosphate mannosyltransferase1
SLC25A5	Solute carrier family 25 member 5
DEDD	Death effector domain-containing protein
DECR1	2,4-dienoyl-CoA reductase 1
ZYG11B	Zyg-11 family member B
THY1	Thy-1 membrane glycoprotein

### VB1 Binds to MAPK1 by Interacting With the Residues E58 and R65

**FIGURE 4:**
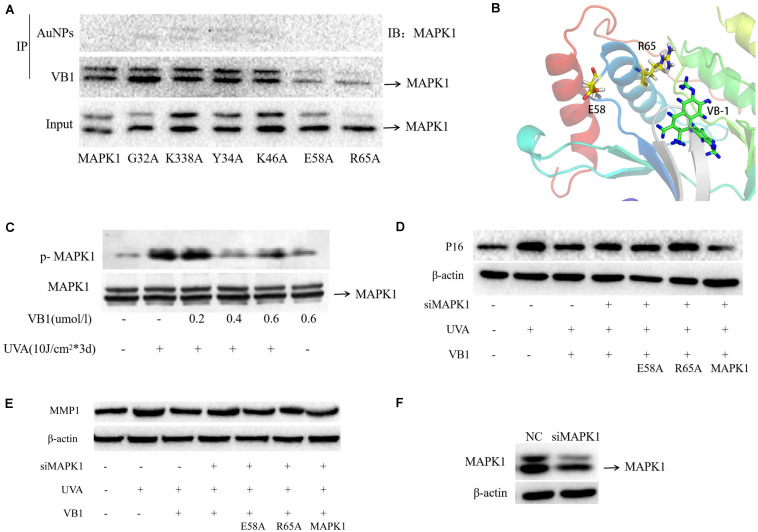
VB1 binds to MAPK1 through E58 and R65 residues of MAPK1 and VB1 protects 293T cells from UVA-induced senescence via binding the two residues. **(A)** VB1 linked with gold nanoparticles was used for the immunoprecipitation in 293T cells. In the gold nanoparticles (AuNPs) immunocomplexes, MAPK1 was not detectable by western blot analysis, while MAPK1 was detectable by western blot analysis in nanogold-VB1-immunoprecipitated complexes. In the 293T cells transfected with E58-mutant MAPK1 and R65-mutant MAPK1, MAPK1 was not detected, while MAPK1 was detectable in cells transfected with other four mutant MAPK1. **(B)** The combination form between VB1 and MAPK1 via E58 and R65 residues using computer-aided methods. **(C)** p-MAPK1 expression was detected by western blot analysis. p-MAPK1 was significantly increased after UVA irradiation and that VB1 could significantly decrease UVA-induced p-MAPK1 expression in a dose-dependent manner. **(D,E)** 293T cells was used in further co-transfected experiments because of difficulty in HDFs. Endogenous MAPK1 in 293T cells was knockdown by MAPK1 siRNA. Then UVA-irradiated 293T cells were co-transfected with VB1 and wild-type or mutant MAPK1 (E58-mutant or R65-mutant), and p16 and MMP1 expression was detected by western blot analysis. UVA-induced p16 and MMP1 were significantly decreased in 293T cells tranfected with wild-type MAPK1 and VB1, while p16 and MMP1 were partially reversed in 293T cells transfected with E58-mutant or R65-mutant MAPK1 and VB1. **(F)** MAPK1 siRNA could significantly knockdown the endogenous MAPK1 expression in 293T cells.

We performed endogenous immunoprecipitation assays to confirm whether VB1 directly binds to MAPK1. HEK293T cells transfected with unlinked (control) or VB1-linked nanogold particles were used for immunoprecipitation, and MAPK1 was then detected by western blotting. The results showed that MAPK1 was detectable in the nanogold-VB1-immunoprecipitated complexes but not in the control nanogold immunocomplexes (Figure 4A), revealing that MAPK1 directly bound to VB1. To precisely identify the interaction sites of VB1 with MAPK1, we established wild-type and mutant MAPK1 vectors and used them to transfect HEK293T cells, together with nanogold-VB1 particles. Then, we determined the interactions between VB1 and wild-type/mutant MAPK1 using immunoprecipitation. MAPK1 was not detected in HEK293T cells transfected with the E58- and R65-mutants of MAPK1, while it was detectable in cells transfected with wild-type MAPK1 and the other four MAPK1 mutants (Figure 4A). Then, we modeled the complex between VB1 and MAPK1, considering the interacting residues E58 and R65, by *in silico* method (Figure 4B). These findings indicated that VB1 bound to MAPK1 by interacting with the residues E58 and R65.

### VB1 Can Partially Reverses UVA-Induced Phosphorylation of MAPK1

Only phosphorylated MAPK1 (p-MAPK1), which is the active form of MAPK1, can activate the activity of a series of downstream transcription factors, thereby regulating cell function. To clarify the effect of VB1 on the MAPK1 pathway, p-MAPK1 was detected in HDFs by western blotting in HDFs. The results revealed that p-MAPK1 expression was significantly increased after UVA irradiation, however, VB1 could significantly reverse this UVA-mediated effect (Figure 4C).

### VB1 Protects HEK293T Cells From UVA-Induced Senescence via Binding to MAPK1

To examine whether VB1 reduces UVA-induced senescence through MAPK1 binding, we co-transfected HEK293T cells, with down-regulated endogenous MAPK1, with VB1 and wild-type or mutant MAPK1 (E58- or R65-mutants) and irradiated the cells with UVA rays. We found that the UVA-induced expression of p16 and MMP1 was significantly decreased in HEK293T cells transfected with wild-type MAPK1 and VB1. In contrast, the UVA-induced expression of these proteins was partially reversed in cells co-transfected with both MAPK1 mutants and VB1 (Figures 4D–F). These data revealed that VB1 could protect HEK293T cells from UVA-induced senescence by binding the E58 and R65 residues of MAPK1.

### Topical VB1 Gel Alleviates the Skin Photoaging Phenotype in Mice

**FIGURE 5:**
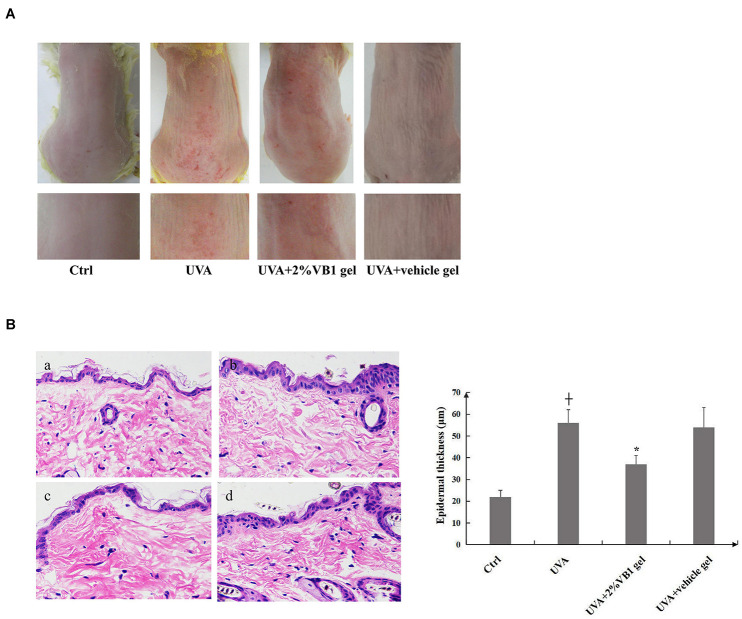
Topical VB1 gel palliates skin photoaging phenotype induced by UVA irradiation in mice. Mice were irradiated with UVA 3 times per week at doses of 20 J/cm^2^ for 12 weeks. A gel containing 2% VB1 or a vehicle gel lacking VB1 (negative control) was applied topically to the dorsal skin areas of mice at 1 h after each irradiation. The control group consisted of untreated mice at the same age. **(A)** Representative photographs of mice dorsal skin in mice from control group, UVA-irradiated group, the experimental group receiving VB1 gel and the vehicle only group. **(B)** Representative photographs of hematoxylin-eosin (HE)-stained dorsal skin sections obtained at the end of the experiment from (a) control group, (b) UVA-irradiated group, (c) the experimental group receiving VB1 gel and (d) the vehicle only group (left panel, magnification 200x). Epidermal thicknesses were estimated at 5 different random sites with each mouse from digital images of HE-stained sections and are depicted as means ± SEM (*n* = 10 per group). The right panel showed the analysis data (+ vs. ctrl, *p* < 0.05, * vs. UVA, *p* < 0.05).

To further evaluate the anti-photoaging ability of VB1, we applied a gel containing 2% VB1 or a vehicle gel lacking VB1 topically on the UVA-irradiated dorsal skin of mice. In the vehicle group, the dorsal skin of the animals was rough and scaly, showing increased thickness and deep wrinkles after 12 weeks of UVA-irradiation compared with the corresponding parameters in the non-irradiated control group. In contrast, the skin conditions of VB1-treated mice were visibly improved, and the formation of skin wrinkles was significantly reduced (Figure 5A). Mouse dorsal skin from each group was harvested for hematoxylin and eosin (HE) staining. The epidermal thickness was markedly higher in the vehicle group than in the non-irradiated control group (Figure 5B). Daily topical application of VB1 gel significantly reduced the thickening of the epidermal layers. These data demonstrated that VB1 could alleviates UVA-induced skin photoaging *in vivo*.

## Discussion

Skin photoaging, an essential aspect of skin aging, is mainly characterized by skin relaxation, wrinkle formation, pigmentation, and telangiectasia, etc. Recent evidence has shown that UVA irradiation produces ROS and induces cell senescence, ultimately leading to skin photoaging (Wlaschek et al., 2001; Yi et al., 2018). Thus, finding the ideal antioxidants that can act as anti-aging drugs is very promising.

Previous studies have demonstrated that VB1 acts as a novel antitumor agent by regulating the cell cycle arrest and apoptosis induction in various cancers. Some studies have shown that VB1 has a strong antioxidant effect and can inhibit multiple protein kinases and signal transduction pathways (Liu et al., 2014, 2018; Yang et al., 2014; Luo et al., 2018). However, the role of VB1 in skin photoaging has never been reported. Here, we showed that VB1 protects HDFs from UVA-induced senescence. Thus, for the first time, the role of VB1 in the skin cellular senescence was explored.

How does VB1 protect HDFs from UVA-induced senescence? To answer this question, we explored potential target proteins of VB1 via mass spectrometry and nanogold-based immunoprecipitation. Nanogold particles, also called gold nanoparticles (AuNPs), have been widely used for the identification of both biological and chemical materials (Lee et al., 2018). When it is combined with recognition proteins, such as antibodies or receptors, this nanomaterial can act as a biosensor molecule (Egea et al., 2019). To date, nanogold particles have been used as a biological tool in many studies, especially in cancer-related studies (Shen et al., 2018). In the present study, nanogold particles were used to pull down VB1 micromolecules for subsequent mass spectrometry and immunoprecipitation analyses. Through mass spectrometry, we identified 26 proteins that potentially bind VB1. Among those 26 proteins, some proteins were tumor-related, such as YWHAQ and eEF1A1, and others were proteins were involved in various biological processes, such as DEDD and MAPK1. The MAPK pathway is one of the most important pathways in aging. It mainly triggers a series of downstream biological effects through MAPK family molecules, including ERK1, ERK2, ERK5, JNK, and p38 MAPK, thereby regulating cell proliferation, differentiation, and development. Some reports suggest that the activation of the MAPK pathway is the central event in UV-induced intracellular signaling, causing nuclear and DNA damage-originated cellular responses (Bode and Dong, 2003). MAPK1, also called ERK2, plays an indispensable role in the MAPK pathway. Only phosphorylated (active) MAPK1 can trigger the activation a series of downstream transcription factors, such as Sata1/3 and FoxO3 to regulate cellular processes, however, it also plays an important regulatory role in aging (Gkotinakou et al., 2019; Zhu et al., 2019). Due to its important role in photoaging, we chose MAPK1 for *in silico* experiments to identify potential VB1-binding sites.

A new computer-aided method, including molecular docking and molecular dynamics simulation, which was widely used to find the “best” matching between two molecules and also can predict their “correct” binding (Akbarabadi et al., 2019; Sakr et al., 2019), was also applied to predict potential binding sites of VB1 in MAPK1. Based on the *in silico* results, we concluded that VB1 could directly target MAPK1 by interacting with several amino acid residues (G32, Y34, K46, E58, R65, and K338). Then, through transfection of HEK293T cells with MAPK1 vectors containing mutant residues and subsequent immunoprecipitation, we discovered that VB1 delayed UVA-induced cellular senescence by binding to the residues E58 and R65. Thus, we hypothesized that VB1 reduced cellular senescence by regulating the expression of phosphorylated MAPK1 expression through direct interaction with MAPK1. Previous studies have shown that TGF-β alone induced Ras-Raf-MEK1 and phosphorylated MAPK1 to increase the expression of MMP1, so we speculate that VB1 could decrease MMP1 and p16 by reducing p-MAPK1 (Amatangelo et al., 2012).

The demand for products that diminish wrinkles and maintain a youthful appearance of the skin is increasing. Currently, all-trans-retinoic acid (ATRA) is the only topical drug that is approved by the Food and Drug Administration (FDA) for the treatment of photoaged skin (Behairi et al., 2016). However, the topical use of ATRA might induce local skin side effects, including irritation, erythema, burning, pruritus, and scaling. Therefore, there is a need for safe and efficacious agents for the prevention and treatment of photoaging. To analyze whether VB1-containing preparations can effectively delay skin photoaging, we prepared a VB-1 gel and demonstrated that the gel exhibited a good permeate rate and low lag time (Li et al., 2016). In addition, topical administration of the VB1 gel to mice remarkably reduced skin photoaging phenotypes caused by long-term UVA irradiation.

In summary, we demonstrated that VB1 significantly inhibits UVA-induced senescence in HDFs by targeting the E58 and R65 residues of MAPK1 and effectively reduces skin photoaging in UVA-irradiated mice, indicating that VB1 could serve as a novel agent for the prevention and the potential treatment of photoaging.

## Data Availability Statement

All datasets generated for this study are included in the article/Supplementary Material.

## Ethics Statement

The animal study was reviewed and approved by the Clinical Research Ethics Committee at the Xiangya Hospital of Central South University.

## Author Contributions

BW and SY performed all the experiments and prepared the figures and tables. SY, QZ, ZD, YZ, and YY provided with the technical and statistical assistance. BW, HX, and YH analyzed and interpreted the data. JL provided all the theoretical direction. BW, SY, HX, and JL wrote and edited the manuscript. All authors read and approved the final manuscript.

## Conflict of Interest

The authors declare that the research was conducted in the absence of any commercial or financial relationships that could be construed as a potential conflict of interest.

## Abbreviations

ATRA: all-trans-retinoic acid
ERK1/2: Extracellular signal-regulated protein kinases1/2
FDA: Food and Drug Administration
HDFs: human dermal fibroblasts
JNK: c-Jun NH2-terminal kinase
MAPK1: Mitogen Activated Protein Kinase 1
MMP1: matrix metalloproteinase-1
p-MAPK1: Phosphorylated MAPK1
UVA: ultraviolet A
VB1: Purified vitexin compound 1.
