# Supplementary material for: Purified Vitexin Compound 1 Inhibits UVA-Induced Cellular Senescence in Human Dermal Fibroblasts by Binding Mitogen-Activated Protein Kinase 1
Source: Front Cell Dev Biol. 2020 Jul 31;8:691. doi: 10.3389/fcell.2020.00691 (PMC7413062; doi:10.3389/fcell.2020.00691)
Supplement: Supplementary file 1 [file Table_1.DOC]

**Supplement table The primers used for the MAPK1 mutants**

| **Amino acid residues** | **The sequences of primers** |
| --- | --- |
| MAPK1-G32A(F) | GAGGCCGCCTACGGCATGGTGTGCTCTGCTTATGATAATGTC |
| MAPK1-G32A(R) | CATGCCGTAGGCGGCCTCGCCGATGTACGAGAGGTTGGTGTA |
| MAPK1-Y34A(F) | GAGGGCGCCGCAGGCATGGTGTGCTCTGCTTATGATAATGTC |
| MAPK1-Y34A(R) | CATGCCTGCGGCGCCCTCGCCGATGTACGAGAGGTTGGTGTA |
| MAPK1-K46A (F) | GTCAACGCAGTTCGAGTAGCTATCAAGAAAATCAGCCCCTTTGA |
| MAPK1-K46A (R) | GCTACTCGAACTGCGTTGACATTATCATAAGCAGAGCACACCATG |
| MAPK1-E58A(F) | CCTTTGCACACCAGACCTACTGCCAGAGAACCCTGAGGGAGA |
| MAPK1-E58A(R) | TAGGTCTGGTGTGCAAAGGGGCTGATTTTCTTGATAGCTACTC |
| MAPK1-R65A(F) | CTACTGCCAGGCAACCCTGAGGGAGATAAAAATCTTACTGCGCTT |
| MAPK1-R65A(R) | TCAGGGTTGCCTGGCAGTAGGTCTGGTGCTCAAAGGGGCTGATT |
| MAPK1-K338A(F) | GGATGACTTGCCTGCAGAAAAGCTCAAAGAACTAATTTTTGAAG |
| MAPK1-K338A(R) | TTCTGCAGGCAAGTCATCCAATTCCATGTCGAACTTGAATGGTG |
